# Climate warming drives pulsed resources and disease outbreak risk

**DOI:** 10.1098/rspb.2025.1340

**Published:** 2025-08-13

**Authors:** Rémi Fay, Marlène Gamelon, Thibaud Porphyre

**Affiliations:** ^1^ Laboratoire de Biométrie et Biologie Évolutive, Unité Mixte de Recherche (UMR) 5558, Université Claude Bernard Lyon 1, CNRS, VetAgro Sup, Villeurbanne, France

**Keywords:** climate change, disease ecology, host–pathogen interaction, infectious disease, population dynamics, masting

## Abstract

Climate influences the risk of disease transmission and spread through its direct effects on the survival and reproduction of hosts and pathogens. However, the indirect influences of climate variation (i.e. those mediated by food resources on host demography) are often neglected. Pulsed resources produced by oak trees in temperate forests constitute important resources for seed consumers and strongly depend on temperature. Using an individual-based model, we provide a theoretical exploration of the influence of climate warming on the dynamics of the African swine fever (ASF) in the seed consumer wild boar (*Sus scrofa*), considering both direct and indirect temperature effects. We show that climate warming directly decreases the persistence of the virus in the environment, but also increases the production of acorns, with cascading effects on the seed consumer host species. Integrating these climatic effects suggests a decrease of ASF spread under future warmer conditions. Importantly, food-mediated indirect effects of climate may outweigh direct effects, reversing, in some situations, the predictions of epidemic dynamics under climate change. This shows that anticipating future epidemic risks requires a deep understanding of ecological systems, including all direct and indirect climatic effects.

## 1. Introduction

Climate warming is anticipated to alter many ecosystem processes, including the dynamics of infectious diseases. Since temperature optima generally differ between hosts and pathogens, rising temperatures are likely to shift the nature of their interactions [1,2]. Predicting how climate change will affect host–pathogen dynamics is critical for developing effective prevention strategies to reduce future epidemic risks. However, this remains challenging as temperature influences these dynamics both directly and indirectly.

Temperature directly influences the demographic rates of hosts and pathogens (i.e. their survival, reproduction and infectiousness [3–5]). As a result, interactions between hosts and pathogens are likely to be altered, thereby changing the risk of disease spread and epidemic outbreak. Many studies have assessed the direct effects of temperature on hosts and pathogens, determining, for instance, thermal performance curves in experimental studies [6,7]. Host–pathogen dynamics can also be influenced by indirect temperature effects, particularly those that are mediated by food resources. Changes in food availability can impact host demographic rates [3,8], movements and aggregation [9], and immune defences by strengthening or weakening allocation trade-off among competing physiological functions [10]. Temperature effects on food resources could be a major route, especially for homeotherm hosts such as mammals, which are likely to be less vulnerable to direct temperature effects than ectotherms [11]. Although they may be important drivers in disease spread, indirect temperature effects are often overlooked when assessing future risk of disease epidemics in natural populations.

Some pulsed resources produced by plants, such as acorns produced by oak trees, are driven by temperature, and are thus expected to change under climate warming [12,13]. These mast events, which lead to strong annual variation of seed or fruit abundance over large areas, have a strong impact on animal consumers, being potential reservoirs for disease (e.g. rodents and wild boar) [14]. Therefore, a system involving a pathogen and a pulse resource consumer as hosts constitutes an excellent case study to investigate direct and indirect effects of temperature on epidemic dynamics in natural populations.

**Figure 1.**
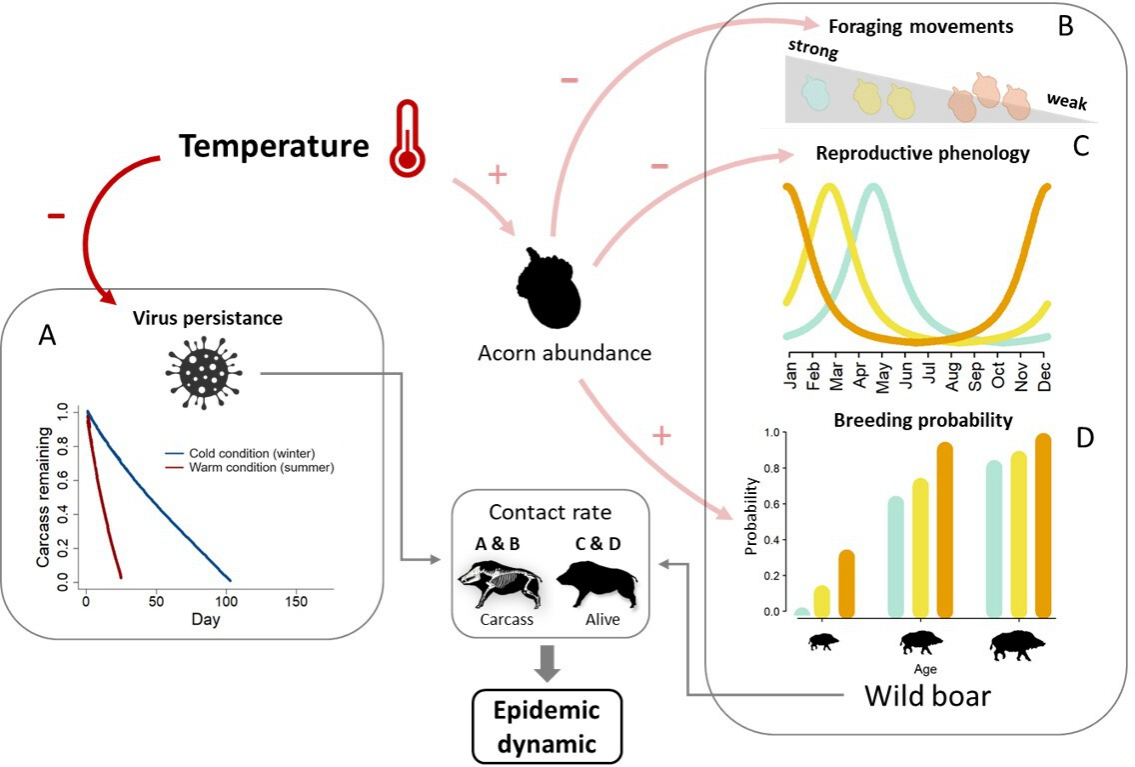
Overview of the influence of the temperature on the wild boar and African swine fever (ASF) dynamics. Dark red arrows figure direct temperature effects on epidemic dynamic and light red arrow figure indirect temperature effects mediated through acorn production, with the sign indicating the positive or negative influence of temperature. (A) Carcass degradation as a function of temperature driving virus persistence in the environment (illustration for a 40 kg carcass with average winter and summer temperature). (B) Foraging movements according to acorn abundance (blue = low, golden = medium and orange = high). (C) Reproductive phenology (birth peak) according to acorn abundance. (D) Variation in age-specific breeding probability according to acorn abundance. All these processes affect contact rates, both among living individuals and with carcasses, impacting epidemic dynamics.

Here, we assess how climate warming directly and indirectly influences host–pathogen dynamics using the study case of wild boar *Sus scrofa* and African swine fever (ASF), a highly contagious viral disease that is currently spreading worldwide. The virus can spread through contacts among living wild boars and with infected carcasses [15]. In this system, increasing temperatures have direct negative effects on virus persistence in the environment [16], and indirect effects on wild boar, through positive influence on oak masting [13], a key food resource for this host species. High acorn production decreases foraging movements [17] (and thus opportunities to contact carcasses [18–20]), advances wild boar birth peak [21] and increases female breeding proportion [22] (figure 1). The magnitude and timing of birth peak may have strong effect on disease dynamics by affecting population density and age structure. Greater population density increases contact rates among living individuals, thereby enhancing the transmission of pathogens, and younger individuals could be more vulnerable to pathogens due to the immaturity and naive state of their immune system [23,24].

Capitalizing on a long-term individual monitoring of a wild boar population in France, along with temperature and acorn production records from the same area, we developed a spatially implicit stochastic individual-based model (IBM) to describe the daily interplay between wild boar population dynamics and ASF transmission dynamics. Our study population is free from this virus and thus epidemic dynamics were investigated using simulations, with virus introduction at variable months to account for seasonal variation [24]. We evaluated both direct and indirect effects of temperature on the wild boar–ASF epidemic dynamics, assessing the contribution of each pathway. Then, we projected host–pathogen dynamics under the RCP 8.5 climate warming scenario to anticipate potential epidemic changes in the coming decades.

## 2. Material and methods

### (a) Study system

The wild boar is a large omnivorous, polygynous and sexually dimorphic ungulate widespread in Eurasia [25]. This study focuses on a hunted population monitored since 1982 in the forest of Châteauvillain-Arc-en-Barrois (48°02*′*N, 4°56*′*E), northeastern France. This population occupies forests dominated by oaks (*Quercus petraea*) and lives under climatic condition intermediate between continental and oceanic. The demography of this population is highly seasonal, with a birth peak in spring and a high mortality rate during the hunting season (October–February) [26]. A capture–mark–recapture programme has allowed data on wild boar demographic rates to be collected since the 1980s (electronic supplementary material, table S1). Annual acorn production was measured indirectly by analysing stomach contents of shot individuals. Three categories of acorn production (high, medium and low) were defined depending on the quantity of seeds found in the stomachs (for further details see [27]). Finally, average daily air temperature was measured from 2003 to 2022 on six Météo France weather stations surrounding our study area (<50 km).

The ASF virus is a DNA-virus of the Asfarviridae family which infects suid species only. In 2007, the disease was introduced in Europe; it rapidly spread both westward and eastward, and now covers most of Europe and Asia [15]. Susceptible individuals can be infected through close contact with infectious individuals (nose to nose, saliva and urine) or with infected carcasses that remain in the environment. With a case-fatality rate approaching 100% and no vaccine available, ASF represents a major socio-economic burden, and a threat to food security and biodiversity [15]. While the disease has not yet been detected in France, it has spread from eastern to western Europe in recent years, making incursion events likely to occur in the near future [28].

### (b) Direct and indirect temperature effects on the wild boar–African swine fever system

Temperature has both direct and indirect effects on this system. Temperature directly modulates ASF persistence in the environment by affecting carcass persistence. ASF survives in a carcass as long as it persists, that is for months in cold winter conditions and a few weeks under warm summer conditions [29] (figure 1A). In contrast, while there is no evidence for a direct effect of temperature on wild boar’s demographic rates in our study population, indirect effects exist through oak masting. Oak masting is highly dependent on thermal conditions during the pollen emission period, and warmer spring conditions increase the frequency of mast seeding events (i.e. massive fruit production spatially synchronized among trees) [13]. Acorn constitutes the preferred food resources for wild boars, and their abundance influence their foraging movements. In particular, when acorn abundance is low, wild boars increase their foraging movement [17], and thus their probability to meet and contact a carcass (figure 1B). High acorn abundance also increases the breeding probability of females (figure 1D, [22,30]) and leads to the advance of their reproductive phenology (figure 1C) [21], but does not affect survival probabilities [22,30]. While increasing food abundance may improve body condition, which may increase immune defence and survival [8], ASF is a highly virulent pathogen and we assumed that immune system efficiency is unlikely to be changed, as suggested by the high mortality rate of captive infected individuals which enjoyed ad libitum food [31].

### (c) Modelling framework

We expand a stochastic IBM previously developed to describe the daily interplay between this wild boar population and ASF transmission dynamics [24]. A detailed presentation of the model is provided in the electronic supplementary material. Briefly, the model is composed of two major components, representing (i) the demographic dynamics of the host population and (ii) the transmission dynamics of the virus within the population. Hosts were modelled individually and characterized by sex, age and health status. Each day, individuals may successively survive or die, breed or not, and change health status. The infection dynamics in the population were modelled considering an SEIR epidemiological process, in which individuals are either susceptible (S), exposed but not infectious (E), infectious (I) or recovered (R) (electronic supplementary material, figure S1). Individuals who died overtime, either from natural causes or from the disease, remain as carcasses in the environment. As such, susceptible individuals can be infected through contacts with living infectious individuals or with infected carcasses. All animals killed during the hunting season were removed from the population and thus do not participate in the disease transmission dynamics. Finally, this IBM is not spatially explicit, and we assume that we modelled a localized and closed wild boar population, corresponding to situations where a population has been isolated following disease detection, as is the case in Europe [15].

#### (i) Wild boar population dynamics

Age- and sex-specific survival and reproductive rates were obtained from the long-term monitoring of the study population (electronic supplementary material, table S1 for parameter values). Reproduction was decomposed in three successive steps: the breeding probability, which was modelled using a Bernoulli distribution, the litter size, which was modelled using a multinomial distribution and the balanced piglet sex ratio, which was randomly determined with a binomial distribution. Females may reproduce from subadult age and once a year at maximum, regardless of their age. Daily breeding probabilities were derived from the annual breeding probabilities and the birth distribution. Birth distribution was modelled based on observational data using a Student distribution (see electronic supplementary material, appendix S1). Both breeding phenology and breeding probabilities were influenced by acorn abundance, distinguishing three set of parameters corresponding to the three levels of acorn production (low, medium and high) (figure 1; electronic supplementary material, table S1). We modelled the positive influence of acorn abundance on earlier birth dates by shifting the birth peak following previous findings in that population [21]. We did not consider the influence of acorn abundance on other aspects of the birth distribution.

#### (ii) Disease transmission and dynamics

Infection risk was modelled using a binomial distribution, where the number of trials corresponds to the number of infectious contacts, and the success probability corresponds to the risk of infection per infectious contact. The daily number of contacts with living individuals was modelled as a function of population size (density dependent), age and sex using a generalized logistic model. The number of contacts with carcasses per individual was frequency dependent and decreased with acorn abundance (figure 1B), hence reflecting the preference of wild boars for acorn and their increasing foraging activity when this resource is rare [17,18]. Quantitative measurements of these changes are nonexistent in the literature. Thus, to be conservative, we parametrized our model using a modest decrease of carcass attractivity with acorn abundance. We assumed that contacts with carcasses were four times less frequent when acorn abundance shift from low to high (electronic supplementary material, table S1).

Once infected, individuals become infectious after an incubation period of four days and remain infectious for an average of 5 days [31]. Most infection events (95%) are fatal, regardless of age, sex and the period of the year [31]. However, if an infected individual survives infection, it transits to the recovery class. Recovered individuals are assumed to be immune for life and cannot be infected again nor can it be infectious. We further assumed no maternal immunity transfer from immunized sows to newborn piglets because no study has yet reported such transfer for virulent strains.

#### (iii) Model initialization and run

Each simulation run was started assuming a population of 100 individuals, mimicking a small and localized wild boar population. Initial age and sex distributions were attributed according to the stable age and sex distribution. All individuals were immunologically naive (susceptible). Host population was projected 1 year prior to the introduction of the virus to ensure independence from the initial conditions. The virus was introduced in the second year in the population by adding one infected sub-adult male (age = 1.5 years), illustrating the immigration of an infected individual within the population. Because the timing of virus introduction has strong influence on the subsequent epidemic dynamics [24], each model was run 12 times introducing the virus on the first day of each month. Following virus introduction, the population was projected for two additional years, leading to a total projection length of 4 years. This projection duration allowed to model the full epidemic dynamics until virus extinction. Each day, we observed the host population size and composition (age, sex and health state), and the number of carcasses, including their infection status. To describe the epidemic dynamics, we computed the basic reproduction number (*R*_0_, the mean number of secondary cases generated by a given infectious case in an entirely susceptible population), the invasion success (i.e. the probability single incursion generates outbreaks of 5 or more cases), Then, given invasion success, we computed the maximum daily incidence rate (i.e. maximum number of new cases observed in a single day) and epidemic duration.

### (d) Assessing temperature effects on the ASF–wild boar system

#### (i) Epidemic dynamics under current climatic conditions

We characterized the epidemic dynamics under current conditions based on temperature and acorn production time series observed from 2003 to 2022. We first repeatedly ran the model for all potential sequences of 4 consecutive years for temperature and corresponding acorn abundance. These models allow us to depict the influence of inter-annual temperature variation on epidemic dynamics, integrating both direct and indirect temperature effects.

Then, we ran additional models to disentangle the direct and indirect effects of temperature. To evaluate the direct effect of temperature (i.e. effect on carcasses persistence), we ran the model for all potential sequences of 4 consecutive years of temperatures, while randomly sampling acorn abundance sequences from the observed series to control for indirect temperature effects (i.e. effect mediated by acorn abundance). To assess the indirect effects of temperature on epidemic dynamics, we ran three models corresponding to the three levels of acorn abundance, while using the average daily temperature variation to control for the direct temperature effects. Because indirect effects operate through three pathways (i.e. foraging movement, reproductive phenology and female breeding probability; figure 1B–D), we also ran models including only one pathway at a time to assess their relative effects. Inactivated pathways were fixed to their value corresponding to the most frequent acorn abundance (low level).

#### (ii) Epidemic dynamics under future climatic conditions

Expected future temperatures in the study area were projected until 2100 using five alternative climatic models from the EURO-CORDEX initiative [32], assuming the RCP 8.5 scenario of future greenhouse gas emission. This ‘business as usual’ scenario is the most pessimistic, leading to end-of-century warming outcomes from 3.3°C to 5.4°C (5th to 95th percentile), with a median of 4.5°C. While this scenario may overestimate gas emission by 2100, its predictions match current emissions and are judged realistic for the midcentury [33]. Acorn projections corresponding to these temperature projections were obtained using a mechanistic resource budget model linking temperatures to oak production [13,30]. Expected acorn production, first obtained on a continuous scale, was discretized according to the three levels of acorn abundance (i.e. low, medium and high). Predictions uncertainly was accounting for by considering 500 alternative acorn abundance time series. Finally, we projected the wild boar–ASF system under expected future climate conditions considering two time windows: 2050−2069 and 2080−2099. This allowed us to project this system under a lower and upper range of warming. We ran the model using all simulated sequences of temperatures and acorn abundance and compared the average epidemic dynamics with estimates obtained from the current climatic conditions. As mentioned earlier, we ran additional models to disentangled the contribution of direct and indirect effects of temperature in the future climatic conditions (electronic supplementary material, table S2).

### (e) Model outputs

The model was built and run using R statistical software v. 4.2.2 [34]. Each scenario was run multiple times to observe 1000 successful virus invasions, allowing us to describe the epidemic dynamics accounting for stochasticity in model output. To describe the epidemic dynamics, we computed the basic reproduction number (*R*_0_), that is the number of secondary infections generated by a single infected individual. Values of *R*_0_ above one indicate that pathogen will grow and generate outbreaks, while values of below suggest the opposite. We also estimated the invasion success, here defined as the probability that at least five individuals were infected. Then, given invasion success, we computed the maximum daily incidence rate (i.e. maximum number of new cases observed in a single day) and the epidemic duration.

## 3. Results

**Figure 2.**
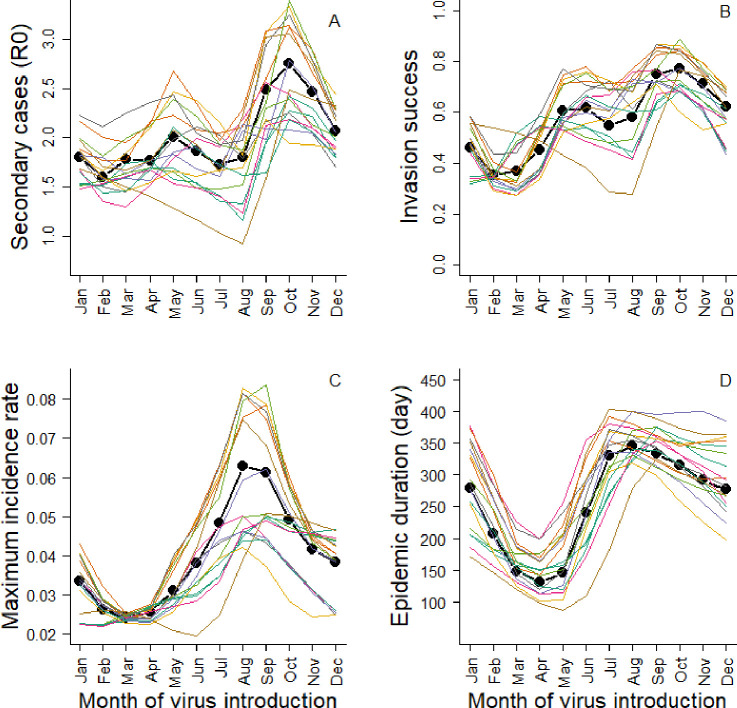
Epidemic dynamics variation generated by interannual variation in temperature between 2003 and 2022 (coloured lines) (both direct and indirect effects of temperature are considered). The bold black line shows the epidemic dynamic under the average environmental conditions. Results are shown for (A) the basic reproduction number (*R*_0_), (B) the invasion success probability defined as the probability that there were at least five secondary cases, (C) the maximum daily incidence rate and (D) the epidemic duration conditional on successful virus invasion. Results are presented as a function of time of virus introduction in the population from 1 January to 1 December.

**Figure 3.**
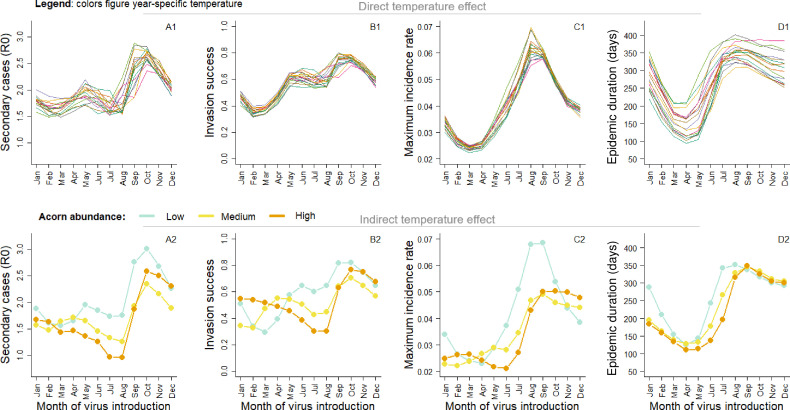
Direct (i.e. through virus persistence in the environment, panel 1) and indirect (i.e. through acorn abundance, panel 2) effect of temperature on epidemic dynamic. On upper panel, each coloured line shows the prediction for specific temperature conditions observed over 4 consecutive years. Results are shown for (A1,A2) the basic reproduction number (*R*_0_), (B1,B2) the invasion success probability defined as the probability that there were at least five secondary cases, (C1,C2) the maximum daily incidence rate and (D1,D2) the epidemic duration conditional on successful virus invasion. Results are presented as a function of time of virus introduction in the population from 1 January to 1 December.

Interannual variation of temperature generates strong variation in epidemic dynamics. For a given month of virus introduction, basic reproduction number varied from 1.6 to 3.1, the invasion success varied from 0.28 to 0.77, the daily maximum incidence rate varied from 0.04 to 0.08 and epidemic duration varied from 111 to 356 days (figure 2; electronic supplementary material, table S3). While both direct (via carcass persistence) and indirect (via food resource) temperature effects generate this interannual variability, direct temperature effects contributed less to the observed variation in epidemic dynamics than indirect effects (figure 3; electronic supplementary material, figure S2). Including only direct or indirect temperature effect led to a mean variation of 6% versus 14% for *R*_0_, 4% versus 15% for invasion risk and 3% versus 15% for the maximal incidence rate (electronic supplementary material, table S3). The only exception was for the epidemic duration that tended to be more affected by the direct effects than by indirect effects (mean variation: 16% versus 11%, electronic supplementary material, table S3). Influence of temperature on epidemic dynamics varied seasonally according to the timing of virus introduction, especially for the maximum incidence rate that was more affected by temperature when the virus was introduced in summer and autumn (June–November, mean variation: 20%) than in winter and spring (December–May, mean variation: 12%; figure 2; electronic supplementary material, table S3). Finally, disease seasonality showed moderate variation according to the thermal condition (figure 2). Seasonal pattern of the epidemic dynamics was poorly influenced by direct temperature effects, but more strongly by indirect temperature effects. Under high acorn abundance, virus invasion success was higher and subsequent outbreaks larger in case of late winter virus introduction (February–March) compared with late spring virus introduction (May–June), but the reverse pattern was observed under low acorn abundance (figure 3).

**Figure 4.**
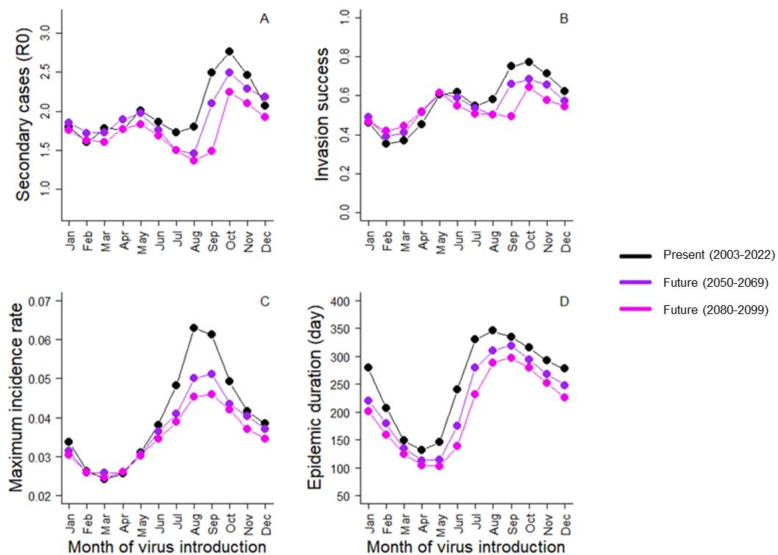
Total influence (i.e. including both direct and indirect effects) of current (black) and future (purple, pink) temperature on epidemic dynamics. Results are shown for (A) the basic reproduction number (R0), (B) the invasion success probability defined as the probability that there were at least five secondary cases, (C) the maximum daily incidence rate and (D) the epidemic duration conditional on successful virus invasion. Results are presented as a function of time of virus introduction in the population from the 1 January to 1 December.

Projecting this host–pathogen system in warmer conditions at the end of the century under the RCP 8.5 scenario, we found that epidemic dynamics were likely to be weaker, with lower *R*_0_ (range of differences according to the month of virus introduction: [+0.03; −1], mean difference: −0.27), lower invasion success (range [+0.07; −0.26], mean: −0.05), lower maximum daily incidence rate (range: [0; −0.017], mean: −0.005) and shorter duration (range: [−24; −102], mean: −54 days) relatively to current temperature conditions (figure 4; table 1). Both direct and indirect effects led to lower epidemic dynamics, but direct temperature effects contributed more to the future decrease in epidemic dynamics than indirect effects (electronic supplementary material, figures S3 and S4). Projecting future dynamics including only direct or indirect temperature effect leads to a mean decline of 13% versus 2% (average of the four epidemic metrics). Importantly, changes in future epidemic dynamics were season-specific (figure 4). Negative changes were stronger when the virus was introduced in summer and autumn (range: −18% to −21% according to the epidemic metric) than in winter and spring (range: −5% to +5%), though epidemic duration remains relatively stable across seasons (table 1). Furthermore, some changes were reversed according to the seasonality. For instance, invasion success was predicted to decrease over summer and autumn condition (−18%), but to increase in autumn and winter (+5%) (table 1).

**Table 1.** Projected epidemic change under future temperature conditions. Estimates were obtained by comparing the epidemic dynamics under present average temperature conditions (2003−2022) to models figuring future climate conditions, including either the influence of temperature through both direct and indirect effects, direct effects only or indirect effects only. To compare present and future temperature conditions, we computed for each epidemic metric the mean difference over the entire year regardless of the time of virus introduction (full year) or over two periods of virus introduction: winter and spring (December–May), and summer and autumn (June–November).

		% change compared with current temperature conditions					
		full year		December–May		**June–November**	
	temperature pathway	2050−2079	2080−2099	2050−2079	2080−2099	2050−2079	2080−2099
*R_0_*	direct and indirect	−5	−13	+3	−5	−11	−21
	direct	−4	−13	−1	−8	−7	−17
	indirect	−1	−3	+5	+5	−5	−9
invasion success	direct and indirect	−3	−8	+4	+5	−9	−18
	direct	−3	−9	−2	−9	−4	−9
	indirect	+0.4	−0.2	+8	+11	−5	−8
maximum incidence	direct and indirect	−9	−13	−1	−4	−13	−19
	direct	−5	−11	−4	−7	−6	−13
	indirect	−5	−6	+1	+2	−8	−10
epidemic duration	direct and indirect	−13	−21	−15	−23	−11	−20
	direct	−11	−20	−13	−22	−10	−19
	indirect	−2	−2	−2	−4	−2	−1

## 4. Discussion

Acorn abundance determines wild boar reproductive phenology [21] with consequences on host–pathogen phenological match and disease seasonality (electronic supplementary material, figure S1). On the one hand, there are higher contact rates and thus pathogen spread among individuals during the birth peak when both population size and the proportion of young individuals peak. On the other hand, pathogen persistence is higher in carcasses at low temperatures. As warmer springs favour high acorn production, push forward the birth peak of wild boar and decrease virus persistence in the environment, the risk of virus invasion decreases and outbreaks are shorter and less intense (figures 3 and 4). Phenological mismatch between consumers and resources has been frequently reported as a consequence of climate change [35], with potential negative impacts for consumer demography [36]. Here, we show that a similar mismatch may occur in a host–pathogen system. Since shifts in host phenology and pathogen seasonality in response to climate change are likely to be frequent [7,37–39], changes in the timing of host–pathogen interactions could be widespread in the wild. Thus, temperature-induced changes in host and pathogen synchrony, rather than changes in the total abundance of host or pathogen alone, can be crucial for determining how temperature change will influence epidemic dynamics [37,39].

In the wild boar–ASF system, indirect temperature effects mediated through oak masting are a major pathway by which temperature influences epidemic dynamics. In other host–disease contexts, fluctuations in acorn abundance positively influence rodent population demography and Lyme disease prevalence [40]. Similarly, changes in grass phenology have been shown affecting elk movements and aggregation, with consequences on brucellosis transmission risk [9]. Indirect temperature effect could be even more complex. In a snail species *Helisoma trivolvis*, warm conditions were found to increase food availability, which, as a consequence, promotes body growth and increases host survival. This improves both host tolerance to infection and parasite (*Ribeiroia ondatrae*) reproduction rate within infected hosts, which lead ultimately to higher parasite prevalence [39]. Changes in food resources under current global warming could thus be an important ecological pathway by which climate influences disease dynamics indirectly in the wild.

Because epidemic responses to climate warming result from the direct and indirect effects of temperature on hosts and pathogens, considering only one component (i.e. host or pathogen, direct or indirect temperature effect) could lead to biased predictions [5]. In the case of ASF, epidemic risk was predicted lower over winter and spring when considering direct effect only (electronic supplementary material, figure S3B), but the opposite was expected after integrating both direct and indirect effects (figure 4B). Similar shift in predictions when integrating or not all temperature effects has been reported in other systems. For instance, in a caterpillar, temperature increases feeding rate and such exposure to a food-mediated pathogen (baculovirus), suggesting an increase of disease prevalence under warmer conditions [41]. Yet increasing temperatures also increases the virulence of the pathogen leading to the quicker death of infected hosts which decrease exposure of susceptible individuals. The latter effect counterbalanced the former, making disease prevalence lower in a warmer environment [41].

Host and pathogen dynamics will inevitably change with climate change, creating potentially new ‘winners’ and ‘losers’ in these interactions [2]. However, depending on the study system, the winner could be either the host [37,39] or the pathogen [5,7]. The output of the new host–pathogen interaction will depend on the system and ecological context, with potentially spatially heterogeneous response for the same host–pathogen system [37]. Overall, predicting the influence of climate change on disease outbreak requires detailed knowledge of the system, emphasizing the value of a deep understanding of ecological systems as a whole.

## Data Availability

Code from this study has been deposited in Zenodo repository [42]. Supplementary material is available online [43].
